# Behavioral and psychological symptoms of dementia in the long-term care setting: assessment of aged adults and intervention for caregivers

**DOI:** 10.1590/1980-5764-DN-2023-0018

**Published:** 2023-12-11

**Authors:** Evelise Saia Rodolpho Duarte, Alessandro Ferrari Jacinto

**Affiliations:** 1 Universidade Federal de São Paulo, Faculdade de Medicina de Botucatu, Departamento de Medicina Interna, Botucatu SP, Brazil.

**Keywords:** Dementia, Caregivers, Aged, Homes for the Aged, Demência, Cuidadores, Idoso, Instituição de Longa Permanência para Idosos

## Abstract

Due to the progressive need for care, older adults with dementia are at risk of becoming institutionalized. Caregivers of these patients are tasked with the challenge of controlling behavioral and psychological symptoms without sufficient knowledge and are therefore at greater risk of developing physical and mental health problems. In this context, psychoeducational interventions can help greatly toward improving caregiver well-being.

**Objective:**

to investigate the prevalence of behavioral and psychological symptoms of dementia (BPSD) in aged residents of long-term care facilities (LTCFs), to determine the prevalence of burden and common mental disorders in caregivers, and to assess the effects of a psychoeducational intervention.

**Methods:**

an intervention study was performed at LTCFs for aged people. The following instruments were used: Self-Reporting Questionnaire and Zarit Burden Interview for caregivers; and the MMSE, Katz Index, Clinical Dementia Rating scale and Neuropsychiatry Inventory-Questionnaire for older adults.

**Results:**

Of the 72 aged residents assessed, 52 (72.2%) were female and mean age was 82.3 (±8.14) years. The most prevalent neuropsychiatric symptoms were euphoria and elation (74%), followed by agitation and aggression (74%). Of the 54 caregivers, 49 (90.7%) were women and mean age was 33.9 (±10.8) years. Overall, 33.3% screened positive for common mental disorder and 36.1% for burden/overload. A statistically significant association was found between burden and working in philanthropic institutions (p=0.003) and also between burden and presence of common mental disorder or otherwise (p=0.001). After the psychoeducational intervention, 42.8% reported reduced burden.

**Conclusion:**

The residents presented neuropsychiatric symptoms. Caregivers showed burden and common mental disorders, especially in philanthropic institutions. It was observed a reduction in burden of caregivers with psychoeducational intervention, showing the importance of this strategy.

## RESUMO

Em razão do aumento progressivo da necessidade de cuidados, idosos com demência apresentam risco de institucionalização. Os cuidadores desses pacientes são desafiados a controlar os sintomas comportamentais e psicológicos da demência sem o conhecimento adequado e, como resultado, estão em maior risco de adoecimento físico e emocional. Nesse contexto, a intervenção psicoeducacional contribui significativamente para a melhoria do bem-estar do cuidador.

**Objetivo:**

Investigar a prevalência de sintomas comportamentais e psicológicos na demência em idosos de instituições de longa permanência, verificar a prevalência de sobrecarga e transtorno mental comum nos cuidadores e avaliar os efeitos de uma intervenção psicoeducacional.

**Métodos:**

Estudo de intervenção realizado em instituições de longa permanência para idosos. Os instrumentos utilizados foram: *Self-Reporting Questionnaire* e *Zarit Burden Interview* para os cuidadores e o Miniexame do Estado Mental, *Katz Index*, *Clinical Dementia Rating* e *Neuropsychiatry Inventory-Questionnaire* para os idosos.

**Resultados:**

Dos 72 idosos, 52 (72,2%) eram mulheres, com média de idade de 82,3 (±8,14) anos. Os sintomas neuropsiquiátricos mais prevalentes foram: euforia e elação (74%) e agitação e agressividade (74%). Dos 54 cuidadores, 49 (90,7%) eram mulheres, com média de idade de 33,9 (±10,8) anos. Apresentaram rastreio positivo para transtorno mental comum 33,3% e para sobrecarga 36,1%. Foi encontrada associação estatisticamente significativa entre sobrecarga e trabalhar em instituições filantrópicas (p=0,003), assim como entre a sobrecarga e ter ou não transtorno mental comum (p=0,001). Após a intervenção psicoeducacional, 42,8% apresentaram redução da sobrecarga.

**Conclusão:**

Os residentes apresentaram sintomas neuropsiquiátricos. Os resultados dos cuidadores indicaram a presença de sobrecarga e outras manifestações em saúde mental, principalmente em instituições filantrópicas, além de demonstrar a importância de intervenções psicoeducativas voltadas aos cuidadores, considerando que foram observados efeitos na redução da sobrecarga para o grupo intervenção.

## INTRODUCTION

Population aging is a global phenomenon accompanied by a rising prevalence of chronic degenerative diseases such as dementia^1,2^.

Dementia is a syndrome characterized by cognitive and behavioral changes which lead to functional decline^3^. Alzheimer Disease (AD) is the most common type of dementia and has a wide variety symptom with loss of functioning that occurs in all cases of dementia, creating dependence on care, albeit in nursing homes or long-term care (LTC)^3,4^.

Due to the steadily increasing physical and mental dependence, as well as the need for professional care, older adults with dementia have a 2-fold greater risk of becoming institutionalized than those without dementia^5,6^.

In this context, caregivers play a key role in maintaining the health of these individuals. Formal caregivers are paid professionals engaged to assist aged people by providing care within the patient’s home or long-term care facilities (LTCFs)^7-9^. Caregivers of individuals with dementia are tasked with controlling the symptoms of the disease without adequate knowledge and faced with multiple challenging tasks^8-10^. As a result, caregivers are at greater risk of impaired physical and mental well-being, as well as overload^10,11^. Therefore, caring for a patient with dementia is associated with a variety of negative consequences for health, particularly when the aged individual receiving care presents neuropsychological symptoms, denoted behavioral and psychological symptoms of dementia (BPSD)^8,9,11^.

The BPSD terminology refers to a constellation of signs and symptoms related to disturbances in perception, thinking, mood or behavior commonly seen in dementia patients^12-14^. BPSD can be grouped into 5 clinical problems: apathy, depression, sleep disturbances, agitation, and psychosis^12,13^. Studies suggest that controlling behavioral and psychological symptoms can be more exhausting for caregivers than managing cognitive decline, making the assessment of these symptoms important^13-15^.

The most commonly observed BPSD are psychotic (hallucination and *delirium*), sleep disturbances, depression, and agitation^16-18^. Pharmacological treatment of BPSD should only be indicated when non-pharmacological measures have proven ineffective^16-18^. Non-pharmacological interventions for BPSD can encompass aged individuals, as well as their family members and caregivers^16-18^.

Psychoeducational intervention is a non-pharmacological approach that can significantly contribute toward improving the well-being of caregivers, including an educational component covering the diagnosis, course, and progression of dementia to help develop competencies for managing individuals with dementia, as well as BPSD^18-20^. Psychoeducation entails a group of actions that involve structured pedagogical materials and resources through a largely informative intervention^19^. Such interventions can yield results by reducing the burden of caregivers and increasing the competencies related to caregiving^20^.

Therefore, the objectives of the present study were to investigate the prevalence of BPSD in aged residents of LTCFs, determine the prevalence of burden and common mental disorders in caregivers, and assess the effects of a psychoeducational intervention.

## METHODS

A study involving LTCFs for older adults with 2 designs was conducted:

- Observational, analytical, and cross-sectional; and
- Experimental. Two definitions for LTCF were used in the present study: private and philanthropic.

The sample included aged residents (age ≥60 years) previously diagnosed with dementia (identified from medical records). Individuals diagnosed with psychiatric disorders other than dementia were excluded.

Caregivers at LTCFs were invited to take part in the study. Inclusion criteria were any educational level, engaged under a paid work contract (non-volunteers), working at a facility for ≥3 months, and agreement to participate in the study after signing the informed consent. Caregivers who were unable to understand the questions contained in the instruments applied were excluded.

The study was approved by the Research Ethics Committee of Faculdade de Medicina de Botucatu (CEP-FMB) under CAAE 85676518.4.0000.5411. The intervention involved caregivers and was, therefore, also registered on the Brazilian Clinical Trials Registry (Registro Brasileiro de Ensaios Clínicos – ReBEC) platform and approved under registration number RBR-7rqxry.

The aim of the psychoeducational intervention was to provide caregivers of aged people with educational content on dementia and training for management of behavioral problems.

Caregivers participated virtually in the 3 lecture modules about aging and dementia, neuropsychiatric symptoms of dementia, and behavioral problems management through links available on YouTube and received informative folders.

Due to the COVID-19 pandemic and consequent ban on visits to the LTCFs, caregivers took part in the online intervention. Of the 56 caregivers assessed in the initial stage of the study, only 31 received the psychoeducational intervention and 14 completed the study. This number of dropouts derived from the loss of subjects during the pandemic and the caregivers initially evaluated were disconnected from the institutions.

The following Instruments were employed to assess the residents:

- Mini-mental state exam (MMSE): screening test for cognitive impairment, comprising questions grouped into 7 categories, each evaluating specific cognitive functions: time orientation, place orientation, registration, attention and calculation, recall, language, and visuospatial ability^21,22^;
- Katz index: measure of functionality of aged adults for independence in performing basic activities of daily living (ADLs)^23^. Divided into 6 domains of ADLs (bathing, dressing, toileting, transferring, continence and feeding) completed by caregivers^23,24^;
- Clinical Dementia Rating (CDR): dementia rating, especially in AD, can be used as a diagnostic instrument to classify the severity of dementia or detecting mild cognitive impairment (MCI)^25^; The Clinical Dementia Rating – Sum Of Boxes (CDR-SOB), which simplifies scoring by summing each domain, was used^25,26^;
- Neuropsychiatric Inventory Questionnaire (NPI-Q): The abbreviated version of the NPI, used to assess BPSD based on information provided by caregivers, was employed^27^. The NPI-Q measures 12 domains of neuropsychiatric symptoms (delusions, hallucinations, agitation/aggression, dysphoria/depression, anxiety, euphoria/elation, apathy/indifference, disinhibition, irritability/lability, aberrant motor behaviors, nighttime behavioral disturbances, and appetite/eating disturbances)^27,28^. Using a Likert scale, the instrument measures the severity of symptoms and fatigue that these symptoms cause to caregivers^27,28^.

The following instruments were employed to assess caregivers pre- and post-intervention:

- Self-Reporting Questionnaire (SRQ-20): screening for common mental disorders (non-psychotic). The SRQ-20 contains 20 straightforward questions with binary responses^29^;
- Zarit Burden Interview (ZBI): assesses the burden on caregivers associated with caring for patients with functional and behavioral disabilities and is the most widely used scale to assess the burden caregivers of dementia patients^30,31^. The instrument comprises 22 items and scores range from 0-4^30^. Higher final scores indicate greater burden^30,31^.

After initial assessment of residents and caregivers, a descriptive analysis of the sociodemographic variables of both groups was carried out. The χ^2^test of independence was applied to determine significant associations between the categorized variables. Spearman’s correlation (*rho*) test was employed to determine correlations between dimension scores on the scales used. MANOVA was applied to explore the relationships between outcomes. Bootstrapping procedures were performed (1,000 resamples; CI: 95% BCa) to obtain greater confidence in results and also to attain a more robust 95% confidence interval for the difference between means^32^.

Post-intervention data for caregivers were analyzed using the Jacobson & Truax (JT) Method^33^. For the analyses, the statistical significance found from pre-test to post-test was used to calculate the Reliable Change Index (RCI). The standard error of difference was calculated using standard deviation and the reliability index of the measuring instrument (Cronbach alpha) from previous studies performed on a representative sample of the population^33^.

## RESULTS

The sample comprised 72 residents, 72.22% (n=52) female and 27.78% (n=20) male, aged 61–98 years (mean age=82.33, SD=8.14 years), and predominantly widowed (62.5%). Overall, 34.72% had 4–7 years of formal education and 31.94% had 8–11 years. AD was the most prevalent type of dementia (62.5%) in both private and philanthropic facilities (71.1 and 48.1%, respectively).

The MMSE was used to determine cognitive function of dementia conditions. On the exam, 93% of the residents scored <18 points — with cut-off defined according to education — indicating cognitive impairment. The KATZ scale was used to assess functional status, where 61% of residents exhibited significant dependence and 36% partial dependence. The CDR scale was applied as a clinical measure of dementia, showing that 50% of the sample had signs of severe dementia, 36% moderate dementia, and 13.8% mild dementia.

**Figure 1.**
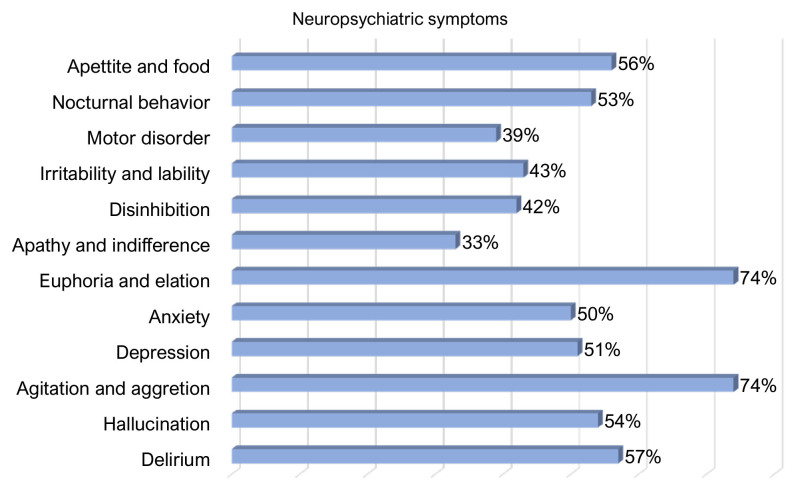
Prevalence of behavioral and psychological symptoms of dementia according to presence of symptom in residents.

The NPI-Q was used to evaluate BPSD in aged participants. The prevalence of each symptom is depicted in Figure 1.

**Figure 2.**
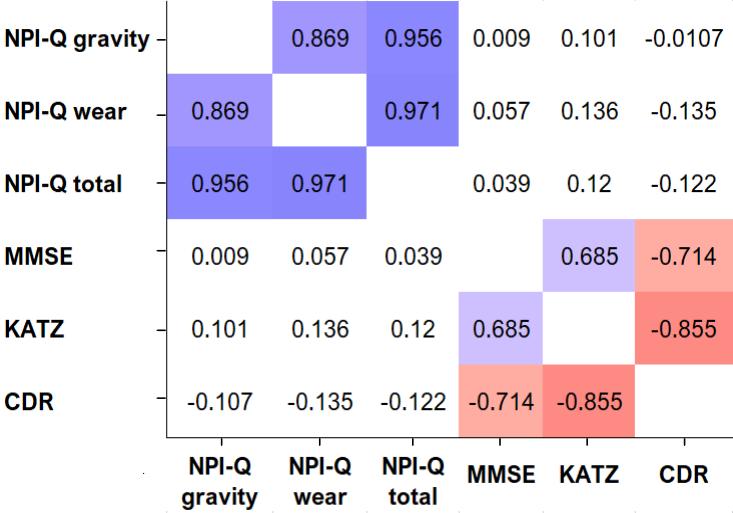
Spearman correlation heat map.

Abbreviations: NPI-Q, Neuropsychiatric Inventory Questionnaire; MMSE, Mini-mental state exam; CDR, Clinical Dementia Rating.

The relationship between neuropsychiatric symptoms (NPI-Q), screening of cognitive function and dementia conditions (MMSE), functional status (KATZ), and clinical assessment of dementia (CDR) of residents was investigated using Spearman’s correlation coefficient (rho). No significant correlation was found between results on the NPI-Q scale and the other variables, with high levels of common variance (94 to 75%). A strong positive correlation was found between scores on the MMSE and the Katz (rho=0.69, n=72, p<0.001), while a strong negative correlation of scores on the CDR scale with both the MMSE (rho=-0.71, n=72, p<0.001) and Katz (rho=0.86, n=72, p<0.001) was detected. The relationship between variables is shown in the correlation heat map (Figure 2).

As for the group of caregivers, 49 (90.7%) were female and age range was 18–60 years (mean 33.9; SD±10.80 years). Overall, 40.7% reported being single. With regard to years of education, the caregivers that reported having 12–13 years and 8–11 of education each represented 42.6% of the sample, where only 3.7% stated having ≤4 years of education. The majority (72%) reported holding only 1 job. For employment setting, 55.6% were engaged at private LTCFs and 44.6% at philanthropic LTCFs and most worked (75.93%) for ≥40 hours per week.

Regarding aspects related to caregivers’ health, the estimates of the presence of common mental disorder (CMD) and burden in caregivers from the two types of facility investigated are given in Table 1.

**Table 1.** Prevalence of common mental disorder and burden by long-term care facility type.

Type of LTC	SRQ-20 categorized	f	%	ZBI categorized	f	%
Private	≥7: with CMD	6	20	With burden	8	26.6
	<7: without CMD	24	80	Without burden	22	73.3
	Total	30	100	Total	30	100
Philanthropic	≥7: with CMD	12	50	With burden	18	75
	<7: without CMD	12	50	Without burden	6	25
	Total	24	100	Total	24	100

Abbreviations: LTC, Long-Term Care; SRQ-20, Self-Reporting Questionnaire; f, frequency; %, relative percentage; ZBI, Zarit Burden Interview; CMD, Common Mental Disorder.

The SRQ-20 was used to screen for non-psychotic mental disorders. Mean score on the SRQ-20 for the overall sample was 5.54 (±4.4), and 33.3% had scores suggesting CMD. By type of facility, 20% of caregivers that worked in private LTCFs exhibited signs of CMD, *versus* 50% of those at philanthropic facilities. The ZBI scale was applied to determine caregiver burden. For the total sample, the mean score on the ZBI scale was 20.98 (±10.69) and 36.11% exhibited burden. By type of facility, 26.6% of caregivers working in private institutions had signs of burden *versus* 75% of those in philanthropic facilities.

In the present study, the chi-square test revealed a significant association between prevalence of caregiver burden and LTCF type, *i.e*., private or philanthropic (c^2^(1)=9.495, p=0.003). An association was also found between prevalence of burden and positive screening for CMD or otherwise (c^2^(1)=12.476, p=0.001).

Multivariate Analysis of Variance (MANOVA) was performed to determine the extent to which levels of non-psychotic mental disorders measured by the SRQ-20 scale and burden measured by the ZBI scale varied for the 2 types of LTCF (private and philanthropic). The descriptive statistics for the groups are shown in Table 2.

**Table 2.** Estimates by group and scale (Self-Reporting Questionnaire-20 and Zarit Burden Interview).

Type of LTC		Mean	Deviation error	f
Score SRQ-20	Private	3.70	3.46	30
	Philanthropic	7.17	4.83	24
	Total	5.24	4.44	54
Score ZBI	Private	15.97	8.83	30
	Philanthropic	27.25	9.55	24
	Total	20.98	10.69	54

Abbreviations: LTC, Long-Term Care; f, frequency; SRQ-20, Self-Reporting Questionnaire; ZBI, Zarit Burden Interview.

The results of the MANOVA revealed a main effect for LTCF type (*F* (2.51)=10.163, p≤0.001; Pillai’s Trace=0.85; *h* ^2^=0.28) on both scales. When analyzed independently, caregivers from philanthropic LTCF had statistically higher scores on the ZBI scale (*M*=27.25) compared to caregivers from private LTCF (*M*=15.97) (*F* (1,52)=20.248; p=0.000). There was a large difference in effect size (*h*=0.28).

Similarly, MANOVA results showed that caregivers from philanthropic LTCF had statistically higher scores on the SRQ-20 scale (*M*=7.17) compared to caregivers from private LTCF (*M*=3.70) (*F* (1,52)=9.408, p=0.003). Again, the effect size was large (*h* ^2^=0.15). Results on the test determining difference in levels of burden and CMD between caregivers working at private *versus* philanthropic LTCF are presented in Table 3, along with the respective confidence intervals via bootstrapping.

**Table 3.** Parameter estimates for SRQ-20 and ZBI by long-term care type.

Variable		Mean difference (I-J)	Error	*t*	Bootstrap^†^	BCa 95% CI		Partial eta squared
					Sig. (2 extremities)	Upper limit	Bottom limit	
Score SRQ-20	Private	-3.467	1..130	-3.067	0.007	-5.684	-1.134	0.153
	Philanthropic	0^*^						
Score ZBI	Private	-11.283	2.508	-4.500	0.001	-15.862	-6.301	0.280
	Philanthropic	0^*^						

Abbreviations: SRQ-20, Self-Reporting Questionnaire; ZBI, Zarit Burden Interview; BCa 95%CI, Bias-corrected and accelerated bootstrap confidencial interval, Si, p-value. Notes: ^*^this parameter is set to zero because it is redundant; ^†^ootstrap results are based on 1000 bootstrap samples.

Ensuing data refers to the post-intervention performed with caregivers in the intervention group. For these analyses, as outlined in the Methods section, the Jacobson-Truax method was used to obtain detailed individualized results for each participant.

SRQ-20 was used to screen for non-psychotic mental disorders. Mean SRQ-20 score in the intervention group was 7.07 (SD=4.04) pre-intervention *versus* 5.71 (SD=4.28) post-intervention. The scores, reliable change index, and status obtained are given in Table 4.

**Table 4.** Estimates of scores, reliable change index and status on Self-Reporting Questionnaire-20.

Participant	Pre	Post	RCI	Status
1	5	0	1.843	AC
2	5	2	1.106	AC
3	6	10	-1.474	AC
4	10	10	0.000	AC
5	10	3	2.580	RPC
6	12	6	2.211	RPC
7	2	5	-1.106	AC
8	11	10	0.369	AC
9	16	14	0.737	AC
10	2	1	0.369	AC
11	4	2	0.737	AC
12	3	11	-2.949	RNC
13	8	3	1.843	AC
14	5	3	0.737	AC

Abbreviations: RCI, Reliable Change Index; AC, Absence of Change; RPC, Reliable Positive Change; RNC, Reliable Negative Change.

**Figure 3.**
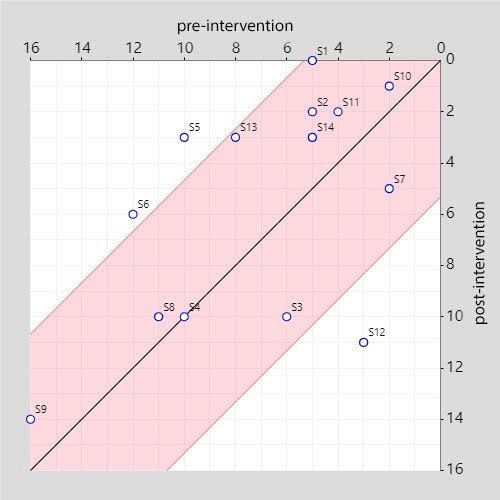
Dispersion of change pre-and post-intervention on Self-Reporting Questionnaire-20.

Participants 5 and 6 showed improvement attributable to the intervention. Participant 12 showed worsening, while for participants 1, 2, 3, 4, 7, 8, 9, 10, 11, 13, and 14, no confirmations of improvement or worsening due to the intervention could be made (absence of change). The corresponding graphic is depicted in Figure 3.

The ZBI scale was applied to determine burden/overload of caregivers of the residents. The mean score on the ZBI scale was 26.85 (SD=8.45) pre-intervention *versus* 20.92 (SD=6.01) post-intervention. The scores and status of participants are presented in Table 5.

**Table 5.** Estimates of scores, reliable change index and status on Zarit Burden Interview scale.

Participant	Pre	Post	RCI	Status
1	15	9	1.291	AC
2	17	17	0.000	AC
3	19	24	-1.076	AC
4	31	17	3.013	RPC
5	34	24	2.152	RPC
6	26	15	2.368	RPC
7	26	23	0.646	AC
8	39	24	3.229	RPC
9	42	23	4.090	RPC
10	23	30	-1.507	AC
11	39	18	4.520	RPC
12	19	33	-3.013	RNC
13	22	16	1.291	AC
14	24	20	0.861	AC

Abbreviations: RCI, Reliable Change Index; AC, Absence of Change; RPC, Reliable Positive Change; RNC, Reliable Negative Change.

**Figure 4.**
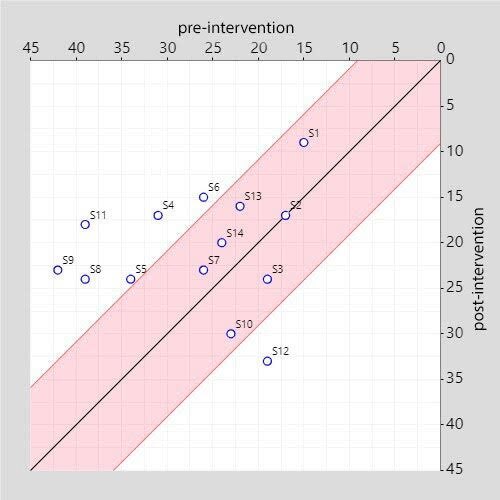
Dispersion of change pre- and post-intervention on Zarit Burden Interview scale.

Participants 4, 5, 6, 8, 9, and 11 showed improvements attributable to the intervention. Participant 12 showed worsening, while for participants 1, 2, 3, 7, 10, 13, and 14, no confirmations of improvement or worsening due to the intervention could be made. The corresponding graphic is depicted in Figure 4.

## DISCUSSION

The results found for the sociodemographic profile of the residents corroborate data from previous studies of the Brazilian aged population, showing greater life expectancy among women (owing to genetic, hormonal, and environmental factors) who, upon living longer, are more likely to be institutionalized^34-37^.

Some studies have characterized the cognitive profile of institutionalized older people and, in general, results show a higher prevalence of signs of cognitive decline among institutionalized individuals^37-39^.

Regarding BPSD, the literature reports rates in LTCF of 75-86%^40,41^. The symptoms which most commonly lead older adults with dementia to be institutionalized are irritability (100%), apathy (80%), verbal aggression (80%), anxiety (74%), depression (54%), agitation (47%), hallucinations (40%), disinhibition and *delirium* (34%)^40-42^.

In terms of the demographic characteristics of caregivers, with respect to gender, the current findings corroborate findings of previous studies showing that the sample comprised predominantly women, irrespective of type of facility (philanthropic or private)^43,44^. The social basis of the role of caregiver may explain the overwhelming female presence among caregivers observed in the majority of studies^43,44^.

Prior to the present investigation, there were no studies in the literature assessing CMDs in this population. However, a study of informal carers of aged adults with dementia found a CMD prevalence of 62.2%, *i.e*., higher than the 20-56% rate reported for the general Brazilian population^45,46^.

A randomized trial exploring information and support entailing 3 stages (assessment, psychoeducation, and training to deal with behavioral problems) reported no change between groups in SRQ-20 performance but found a significant difference in caregiver burden^47^.

Caring for patients with dementia is associated with burden/burnout of caregivers^10,48^. The degree of overload experienced by caregivers depends on a number of factors, such as psychological or emotional health, physical morbidities, social life and income, in additional to neuropsychiatric symptoms of the individual being cared for^48^.

Caregivers from philanthropic institutions demonstrated greater burden when compared to caregivers from private institutions. It is important to consider that, in the municipality studied, the number of aged people assisted by a caregiver in philanthropic LTCF is greater, that is, a caregiver takes care of several aged people. In private LTCF there is a smaller number of older people under the care of caregivers.

Overload and the development of physical or psychic symptoms are commonly presented by those caring for older people with dementia^49^. Post-intervention results in the ZBI showed improvement in terms of reduction in perceived burden. Likewise, a psychoeducational intervention of an informational nature also showed a significant change in perceived burden and in beliefs about caring, showing improvement in the well-being of the caregivers assessed^50^.

Due to the pandemic, the psychoeducational interventional was carried out online and the study had inherent bias due to loss of caregivers who left the LTCF. Additional bias arose because only caregivers present at baseline assessment were selected for the intervention, *i.e*., not all participants selected to form the original sample remained in the study until endpoint. Another limitation of the study was the relatively small sample size of caregivers, where larger samples can yield statistically significant results. The intervention results will be presented in a new article that is currently being prepared.

However, the results revealed the prevalence of BPSD in older residents with dementia institutionalized in the city of Botucatu. Moreover, the findings showed the presence of overload and other mental health problems of caregivers, particularly within philanthropic facilities, while also underscoring the importance of psychoeducational interventions for caregivers in reducing overload. This study proved important in as far as caregivers of aged adults with dementia typically receive no training or guidance on the disease and are unaware of the impact of caregiving on their mental health. Although delivered remotely, the intervention helped improve caregivers’ perceived overload.
